# Citric acid/β-alanine carbon dots as a novel tool for delivery of plasmid DNA into *E. coli* cells

**DOI:** 10.1038/s41598-021-03437-y

**Published:** 2021-12-14

**Authors:** Anju Pandey, Asmita Devkota, Anil Sigdel, Zeinab Yadegari, Korsi Dumenyo, Ali Taheri

**Affiliations:** 1 College of Agriculture, Tennessee State University, 3500 John A Merritt Blvd, Nashville, TN USA; 2 Animal and Dairy Sciences, University of Wisconsin, 1675 Observatory Drive, 266 Animal Sciences Building, Madison, WI USA; 3 Department of Life and Physical Sciences, Fisk University, 1000 17th Ave N, Nashville, TN 37208 USA

**Keywords:** Cell biology, Microbiology, Nanoscience and technology

## Abstract

Successful delivery of plasmid DNA into the microbial cells is fundamental in recombinant DNA technology. Natural bacterial transformation is limited to only certain species due in part to the repulsive forces between negatively charged DNA and bacterial membranes. Most common method of DNA delivery into bacteria is artificial transformation through heat shock and electroporation. These methods require sophisticated instruments and tedious steps in preparation of competent cells. Transformation by conjugation is also not applicable to all plasmids. Nanoparticles have been used successfully in therapeutics for drug delivery into animal cells. They are starting to gain popularity in plant sciences as novel DNA nano carriers. Despite their promise as tool for DNA delivery, their use in microbial cell transformation has not been reported yet. Here we report the synthesis of carbon dots (CDs) from citric acid and β-alanine and their use in DNA delivery into *E. coli* cells. CDs were fabricated using microwave assisted synthesis. Plasmids carrying RFP reporter and ampicillin resistance genes were transferred to bacterial cells and further confirmed using polymerase chain reaction. Our findings indicate that CDs can be used successfully for delivery of foreign DNA of up to 10 kb into *E. coli*. We have demonstrated the use of β-alanine/citric acid carbon dots as nanocarriers of DNA into *E. coli* cells and identified their limitation in terms of the size of plasmid DNA they could carry. Use of these carbon dots is a novel method in foreign DNA delivery into bacterial cells and have a potential for the transformation of resistant organism for which there is still no reliable DNA delivery systems.

## Introduction

Genetic manipulation of an organism is an important aspect of recombinant DNA technology and functional genomics research. These technologies rely on the use of bacterial cells for mass production of the desired proteins and metabolites. Bacterial cells are also used for amplification of the recombinant DNA before insertion into the target organism. Successful delivery of recombinant DNA into the bacterial cell is a challenging task and not all bacteria are amenable to these techniques. The cell envelope of bacteria is composed of negatively charged phospholipids and lipopolysaccharides which repel the negatively charged DNA hence limiting its entrance into the cell. It has been estimated that natural uptake of DNA from surrounding environment occurs in only about 1% bacterial species under laboratory condition^1^. The most common methods for genetic transformation of bacteria are heat shock and electroporation and with both methods, cells must be highly competent to accept the foreign genetic materials from their surroundings. Electroporation utilizes electric current to deliver the plasmid DNA into the competent cell whereas heat shock relies on a combination of chemical treatment (CaCl_2_) and short incubation at high temperature (42 °C). Even though electroporation is reported to be more efficient than heat shock approach, this system requires highly competent cells, specialized apparatus to generate the electric current and cuvette to transfer charge to cells. Also, this system is highly sensitive to presence of salt in the samples and most of the time, ligation reaction samples are needed to be purified before electroporation, in order to avoid sample arching. Hence, with these limitations in bacterial cell transformation, there is a need for alternate DNA delivery methods such as carbon-based nanomaterials. The use of carbon nanoparticles is reported to overcome the drawbacks associated with traditional gene delivery methods including host specificity, transformation complexity, and cells and tissue damage due to applied external forces^2^. Carbon nanomaterials are gaining popularity due to their prominent characteristics including nano structure (1–100 nm), biocompatibility and auto-fluorescence (easy to track inside the cells without staining or tagging with fluorescent molecules)^3^. In addition, they are water soluble and less toxic to the cells and have higher chemical stability and easier preparation steps^4–8^. Therefore, nanomaterial use is becoming popular with applications in different sectors including biotechnology, energy, catalysts, biological labeling, bio-imaging, gene transfer, and drug delivery^6,7,9,10^.

Multiple forms of nanomaterials including, single walled carbon nanotubes (SWNT)^2^, magnetic nanoparticles^11^, mesoporous silica nanoparticles^12^, and carbon dots^13^ have been used in gene delivery. Carbon dots with high chemical stability, auto-fluorescence, customizable surfaces, ease of synthesis and minimal negative impacts on the environment are gaining popularity for gene delivery in humans, animals and even plants^5,6,8,14,15^. However, their use as nanocarriers in microbials transformation has not been reported yet. Here we are reporting the use of carbon dots (CDs) for plasmid DNA delivery into the bacterial cells (*Escherichia coli*) and evaluate their loading capacity in terms of DNA size. We also report the effect of light and temperature on carbon dots potential for plasmid DNA delivery into *E. coli* cells.

## Result and discussion

### Characterization of CDs

Fourier-transform infrared (FTIR) spectroscopy was conducted to characterize the chemical functional groups on the CDs. The prepared CDs showed peaks at 1168 cm^−1^(C–O) of carbonyls group, 1400 cm^−1^ (C–N) of nitrile group, 1693 cm^−1^ (C=O) of ketone group and 2981 cm^−1^ (C–H) of alkane group. The successful passivation of β-alanine is indicated by the presence of 1400 cm^−1^ (C–N stretching)^6,16^. The obtained peaks were in accordance with previous literatures, suggesting successful synthesis of CDs^6,16^.

Photoluminescence properties of CDs synthesized using bottom-up approach, showed two characteristic absorbance peaks at 275 nm and 350 nm. The peak at 275 nm is due to sp^2^‐carbon network and peak at 350 nm is due to n–π* transition of surface carbonyl groups^16,17^. The fluorescence emission profile showed excitation dependent emission spectra of carbon dots synthesized using CA + β-alanine at pH 3. With an increase of excitation wavelength from 335 to 440 nm, the emission peak shifts along with variation in the intensity^16^, giving longer wavelength photoluminescence^18^. Maximum emission peak for CDs is 425 nm upon excitation at 375 nm which is similar to other carbon dots and emits blue fluorescence^16,19^.

CDs synthesized using water at pH 3 had a zeta-potential of −5.46 ± 5.68 mV when measured using Malvern Zetasizer Nano-ZS ZEN 3600^16^. This negative charge is due to the presence of two negatively charged functional groups (C=O and C–O) at the surface of the synthesized CDs which provide sufficient colloidal stability to CDs^20,21^.

### Confocal imaging of the interaction of CDs with bacterial cells

**Figure 1:**
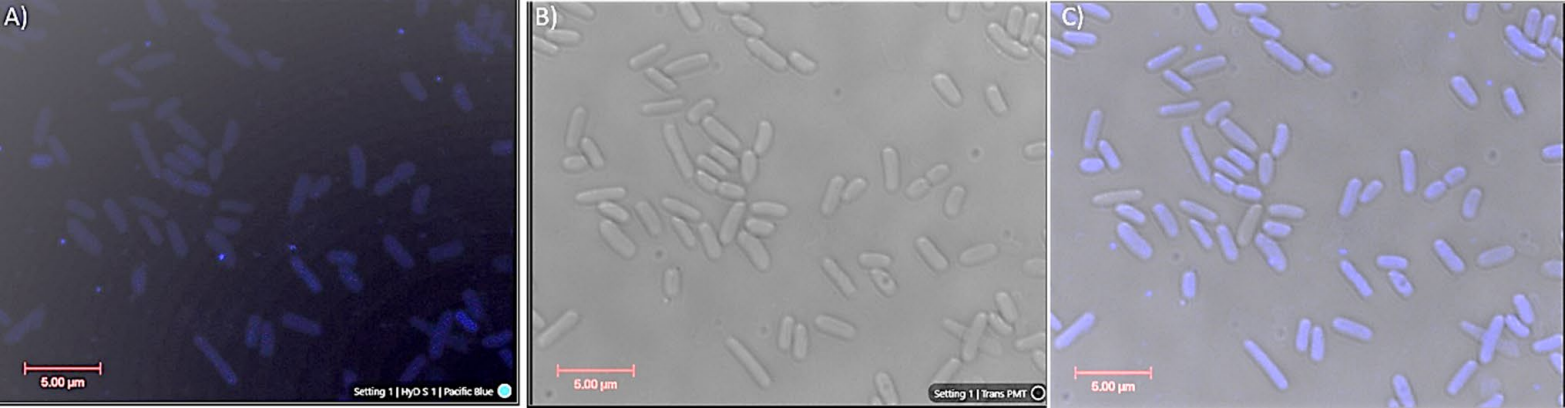
Confocal Microscopy of *E. coli* cells. **(A)** Cells incubated with CA + β-alanine CDs for 15 min under confocal fluorescence microscope **(B)** Cells incubated with CA + β-alanine CDs for 15 min under differential interference contrast (DIC) microscopy **(C)**. Merge visualization of CDs expression and live cells under confocal microscopy (× 63 magnification).

The interaction between cells and CDs was confirmed by confocal microscopy. Five µl of CA + β-alanine CDs (19 mg/mL) (pH3) and 50 µL of bacterial cells (O.D_600_ = 0.5) were incubated for 15 min. The cells were centrifuged and washed three times with sterile water to remove excess carbon dots outside the bacteria. Under confocal microscope, CDs were found bound all over the bacterial cell surface (Fig. 1).

### Delivery of plasmid DNA into *E. coli* cells

In order to find out the effect of pH on functionalization and plasmid delivery of CDs, water with different pH was used to prepare the CDs. *E. coli* (DH5α) cells were mixed with CDs and pU6-pegRNA-GG-Acceptor (RFP) plasmids and incubated for 15 min. Efficiency of CDs in delivery of plasmid DNA into bacterial cells was measured by counting the number of red colonies on LB agar plates. DNA carries a negative charge due to the presence of phosphate group therefore can form electrostatic interaction with positively charged cationic CDs, leading to the formation of CD-DNA complex^22^. Bacterial cells on the other hand, are negatively charged due to the presence of phospholipids, lipopolysaccharides (in gram-negative bacteria) and teichoic acids linked to peptidoglycan (in gram-positive bacteria)^23,24^. The interaction between positively charged CD-DNA complex and negative elements of cell membrane enables them to permeate through cell membrane^22,25^. While inside the cell, carbon nanomaterials release genetic materials in a more controlled and continuous manner due to their minimal cytotoxicity and higher solubility and stability, which leads to a higher transfection rate without damaging the cells^22,26,27^. It has been shown that the amine group on the surface of synthesized CDs helps their DNA load to escape the enzymatic degradation in cytosol that results in continuous DNA release and a higher transformation rate^26^. CDs synthesized using citric acid as a carbon source and β-alanine as zwitterionic surface passivating agent contain both negative and positive surface charges due to the presence of negatively charged carboxyl group and positively charged amine moieties^6^. Therefore, despite their negative surface charge (−5.46), these CDs can successfully deliver DNA into the *E. coli* cells. However, their ability to deliver DNA into other bacteria still needs further investigation.

**Figure 2:**
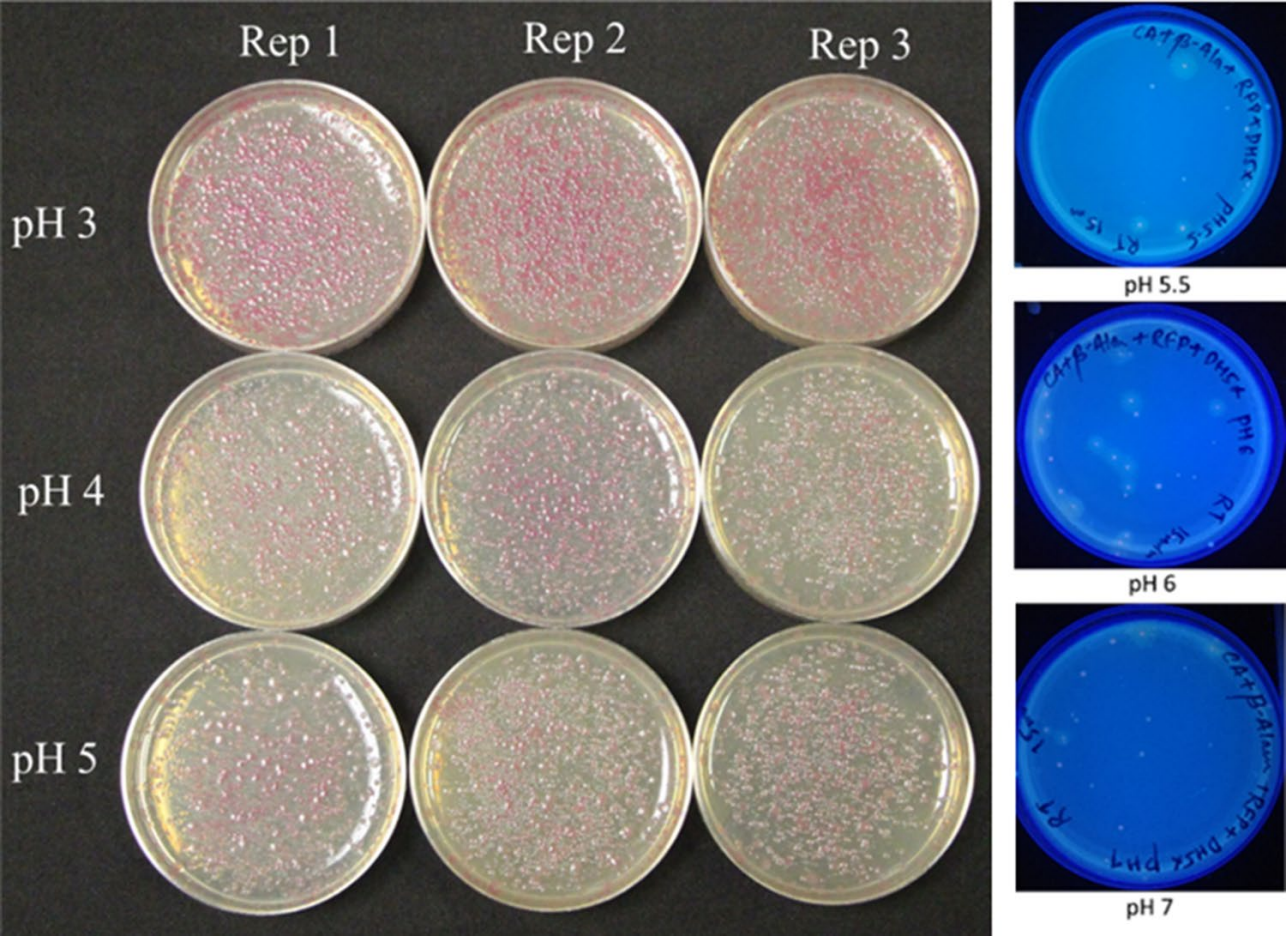
Efficiency of CDs synthesized at different pH for pU6-pegRNA-GG-Acceptor (RFP) plasmid delivery in *E. coli* cells.

We also studied the effect of pH at the time of CDs synthesis on their DNA delivery efficiency. Among different pH used to prepare CDs, pH3, pH4 and pH5 gave a higher number of transformed colonies compared to CDs prepared using pH5.5, pH6, and pH7 (Fig. 2).

**Figure 3:**
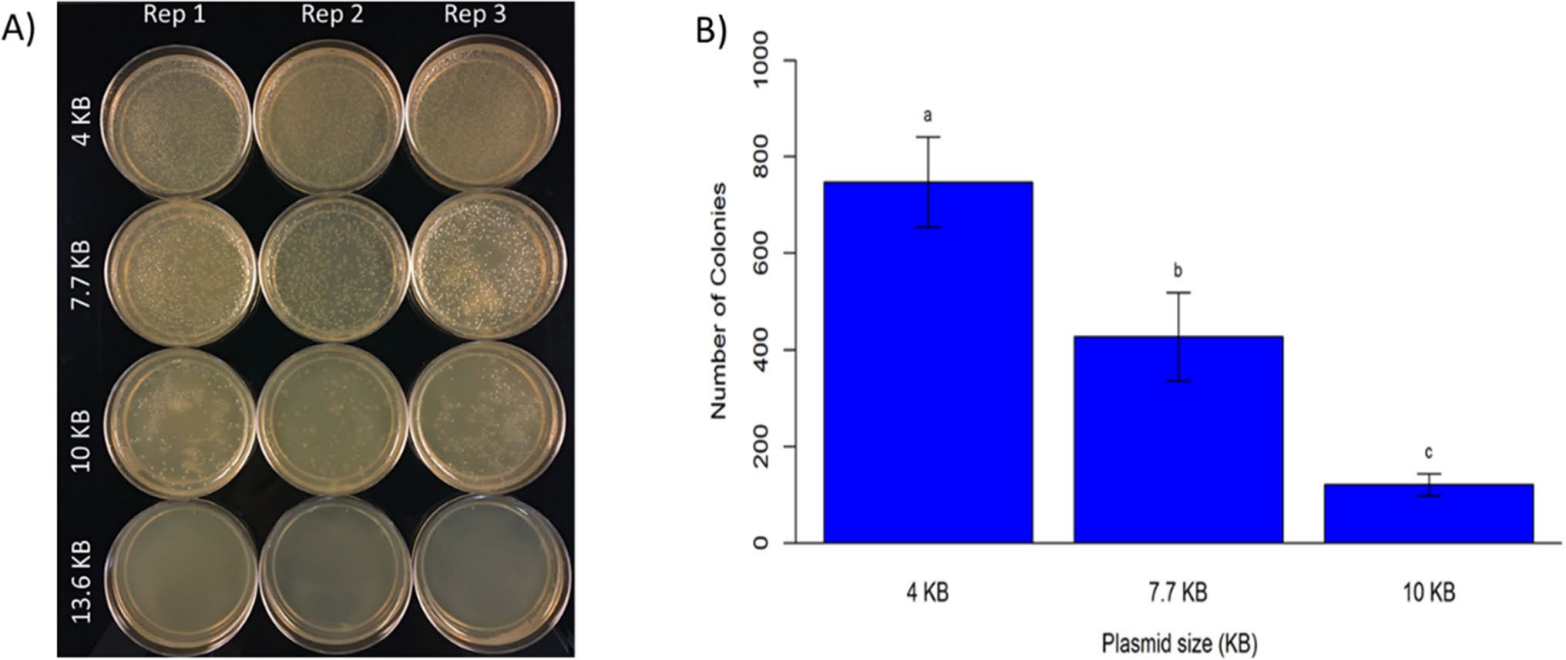
Potential of CDs to deliver the plasmid DNA of different sizes into *E. coli* cells. **(A)** Plates with transformed colonies of *E. coli* using CDs (citric acid + β-alanine) showing number of transformed colonies decreases as the plasmid size increases ranging from 4 to 13.6 kb*.* **(B)** Effect of plasmid size on transformation efficiency. Bar diagram comparison of number of transformed colonies obtained for different size of plasmids. Values are mean from three replicates ± standard deviation.

**Figure 4:**
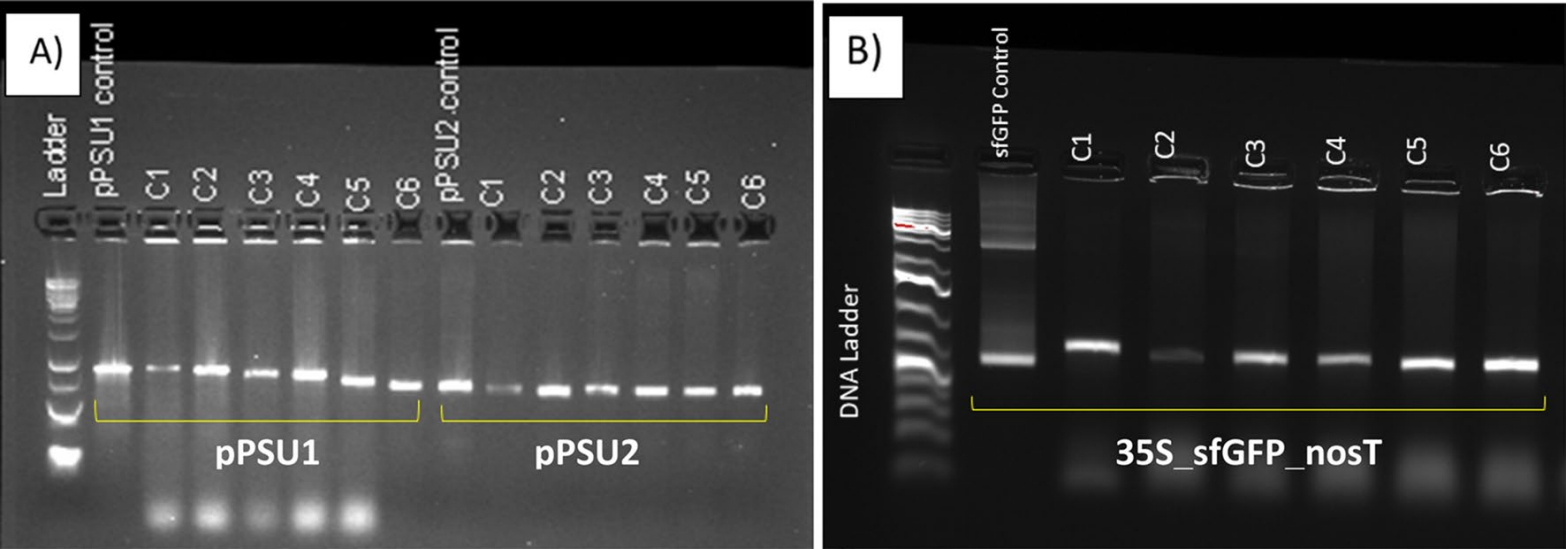
Confirmation of transformation by PCR amplification from positive colonies after plasmid delivery with CA/β-alanine CDs using **(A)** pPSU1 (10 kb), pPSU2 (7.7 kb) and **(B)** 35S_sfGFP_nosT (4 kb) plasmids. Six colonies were randomly picked from each transformation (C1–C6) for colony PCR.

**Figure 5:**
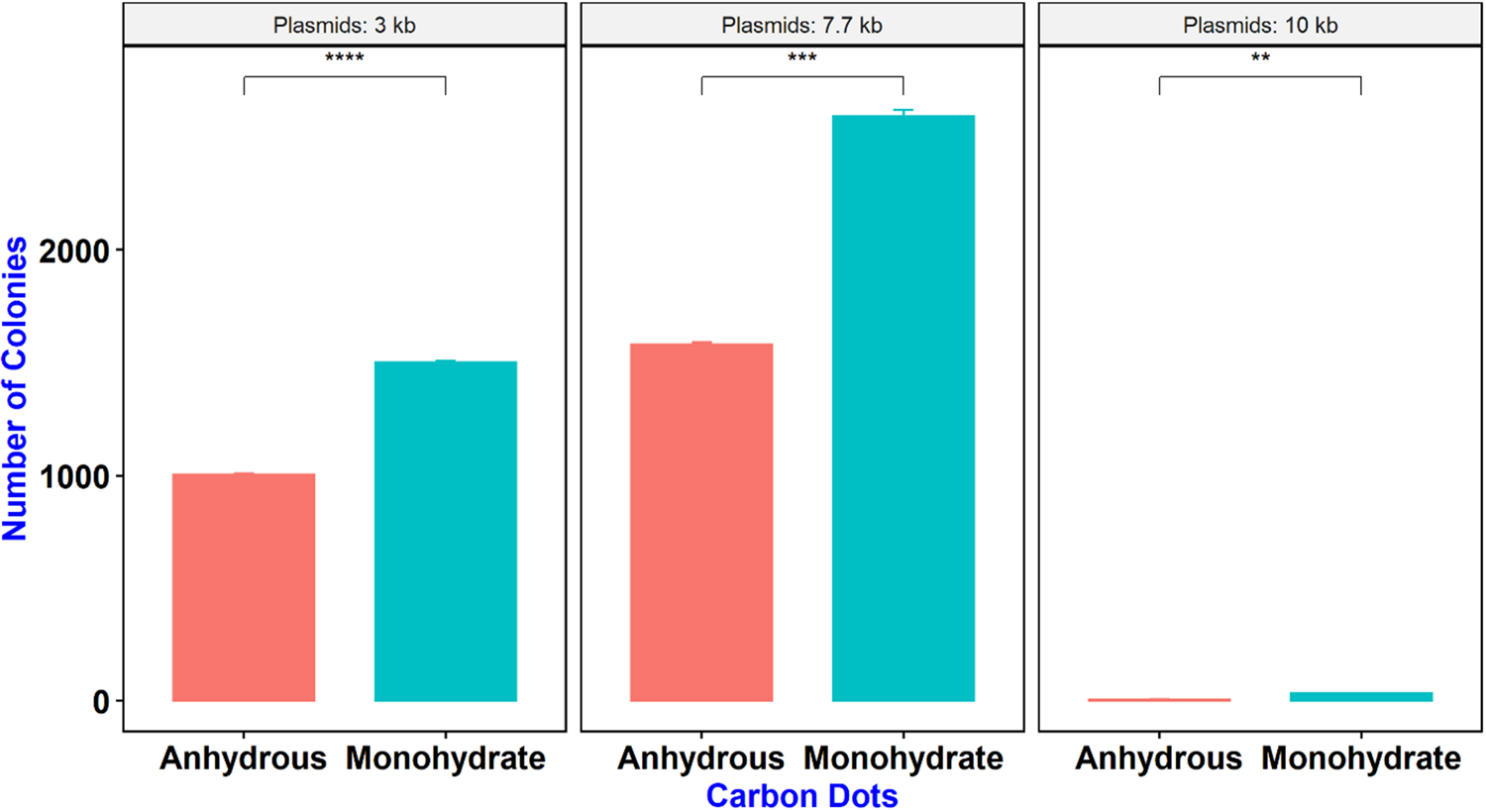
Effect of hydration of CD on transformation efficiency. Bar diagram comparison of the number of colonies obtained from different size of plasmids for anhydrous and monohydrate CDs, indicating better performance of monohydrate CA in DNA delivery. Values are mean (from three replicates) ± standard deviation.

In addition, we tested the maximum size of plasmid that can be delivered into bacterial cells using these CDs. Plasmids with different sizes were incubated with the mixture of bacterial cells and CDs. plasmids that were used for this purpose included: 35Sp_sfGFP_nosT (4 kb), pPSU1 (10 kb), pPSU2 (7.7 kb) and pMDC123 (13.6 kb) (Fig. 3A). Confirmation of delivery was done by colony PCR using M13-Forward and M13-Reverse universal primers (Fig. 4). Our results show that CA + β-alanine CDs was able to deliver plasmids up to a maximum size of 10 kb. However, efficiency for delivery of the 10 kb plasmid was reduced significantly compared to 4 kb and 7.7 kb plasmids (Fig. 3B). Our results also indicate that DNA delivery efficiency is higher when CA monohydrate is used instead of anhydrous CA (Fig. 5).

### Effect of incubation temperature on DNA delivery

**Figure 6:**
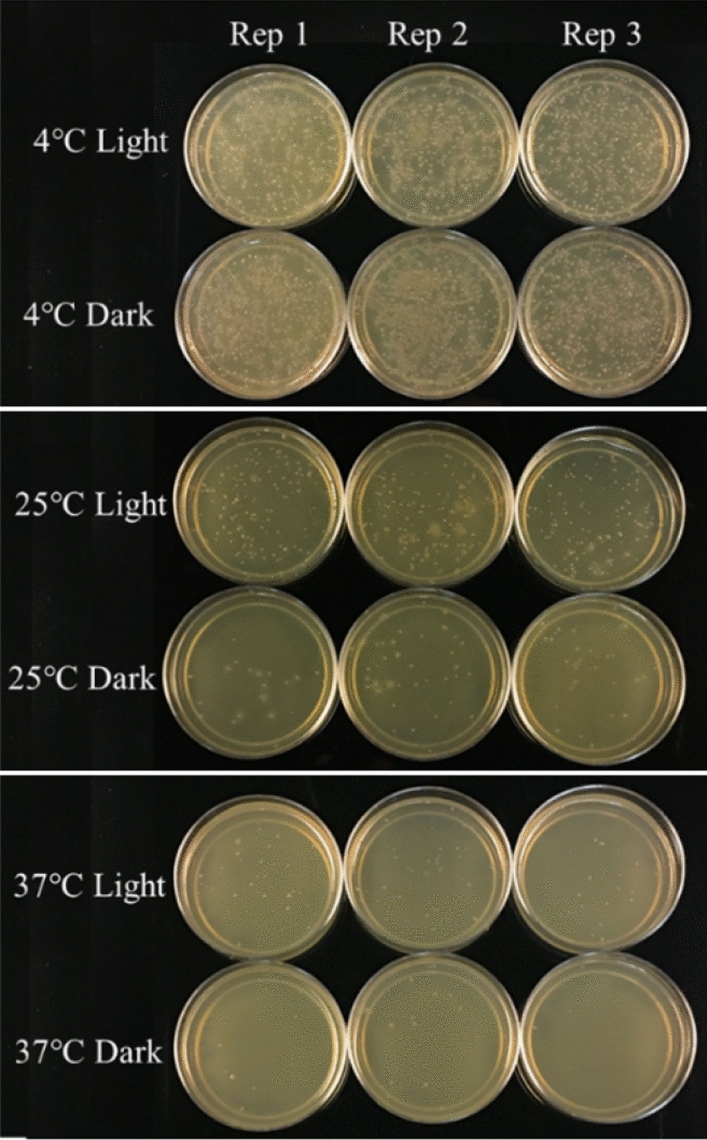
Effect of incubation temperature and light condition on gene delivery by CDs.

**Figure 7:**
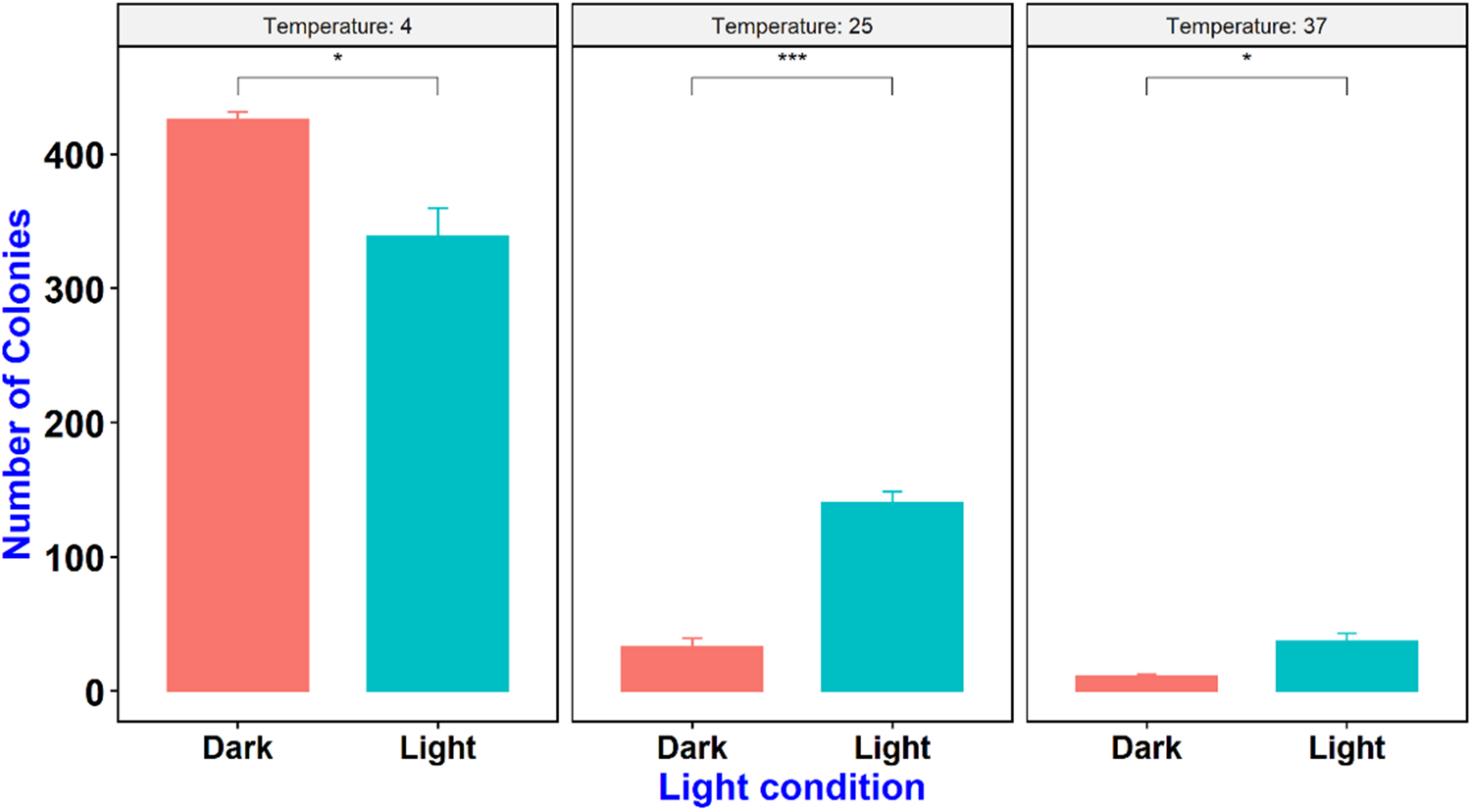
Effect of incubation temperature and light conditions on the number of transformed colonies. Values are mean (from three replicates) ± standard deviation.

An experiment was conducted on the effect of incubation temperature on DNA delivery where the mixture of cells, plasmid, and CDs were incubated at three different temperatures including 37 °C, 25 °C (room temperature) and 4 °C. Our results indicate higher number of transformed colonies in cells incubated at 4 °C followed by 25 °C and 37 °C (Fig. 6) which could be due to the higher activity of *E. coli* cells at higher temperature (37 °C) that leads to higher release of ROS and cell toxicity. Earlier reports also indicate that longer incubation of cells and CDs could lead to antimicrobial activity of CDs which limit their potential as DNA nanocarriers (Fig. 7)^28^. To our knowledge, this is the first report that highlights the effects of temperature on the efficiency of DNA delivery into the cells by carbon nanoparticles.

## Discussion

In this study, we evaluated the efficiency of carbon dots (Citric acid/β-alanine) as novel nanocarriers in delivery of plasmid DNA into *E. coli* cells. The electrostatic interaction between the negatively charge plasmid DNA and positively charged amine group (C–N) at the surface of CDs (Citric acid/β-alanine) facilitate the delivery of plasmid DNA into the bacterial cells. CDs were able to deliver plasmid DNA into *E. coli,* however, the efficiency of gene delivery was dependent on pH of water at the time of preparation of CDs. CDs synthesized at lower pH (pH3-5) had higher efficiency of gene delivery than CDs synthesized at higher pH. This outcome could be the of result of variation in size and number of oxygen functional groups on the surface of CDs. The size of CDs are directly proportional to the pH of water^29^. With lower pH at the time of synthesis, the number of oxygen functional groups increases which results in smaller carbon dots that have better penetrance than larger CDs^6,30,31^. CDs synthesized using leaf extract (*Salvadora oleoides* Decne.) and Zinc nitrate at lower pH (pH 5) were reported to be round in shape with average size of 26.66 nm while CDs synthesized at pH 8 have irregular shape and average size of 38.62 nm^32^. We are planning to perform particle size measurement for CDs synthesized under different pH in the future to confirm the effect of size and shape on their DNA delivery capability (efficiency). In another study, we tested the maximum size of plasmid that can be delivered by these CDs. Our findings indicate that plasmids of up to 10 kb can be delivered into *E. coli* cells using these CDs, however, the efficiency is significantly increased as plasmid sizes decrease (7.7 and 4 kb). Similarly, the effect of incubation temperature on efficiency of DNA delivery was tested by incubating a mixture of cells, CDs and plasmid DNA at three different temperatures (4 °C/temperature that slows down the bacterial growth, 25 °C/room temperature and 37 °C/optimum temperature for bacterial growth). Our findings revealed that incubation temperature affects the transformation efficiency of CDs and higher number of transformed colonies were obtained at 4 °C incubation followed by 25 °C and 37 °C. Outer membrane of bacteria is composed of lipopolysaccharides and their fluidity changes with temperature variations. At higher temperature, membrane fluidity increases which increase the cell membrane permeability. With higher cell membrane permeability, more CDs could enter into the cells and interact with cellular components which leads to higher amount of reactive oxygen species (ROS) and cell toxicity^33,34^. However, at lower temperature, fatty acid tails move closer together and cells become more rigid, reducing the membrane permeability and limiting flow of compounds inside the cells. It is plausible that incubation of cells and CDs at low temperature minimizes ROS toxicity as the cells are not in active growth phase. Further studies are required to measure ROS activity in CD-treated cells under different temperature regimes, to understand the possible reasons why DNA delivery efficiency varies at different temperatures.

## Conclusion

We have demonstrated the prospective of citric acid/β-alanine carbon dots as novel nanocarriers in DNA delivery into *E. coli* cells. We evaluated the effect of light, temperature and DNA size on the efficiency of DNA delivery into *E. coli* cells. Understanding the exact mechanism of DNA delivery into the cells by carbon dots and the effect of temperature on this process needs further investigation. Additionally, further studies are required to evaluate the potential use of CDs in delivery of foreign DNA into other bacterial species.

## Methods

### Materials

All the reagents for CD synthesis were purchased from Thermo Fisher Scientific. Monohydrate citric acid (Cat#A104-500) and β-alanine (Cat#AAA166650I), were used for synthesis of carbon dots. Bacteriological agar (IBI Scientific), Yeast extract (IBI Scientific), Tryptone (MIDSCI™) and NaCl (MIDSCI™) were used to prepare Luria Broth and agar media for *E. coli* culture.

### Synthesis of carbon dots (CDs)

Carbon dots were synthesized using citric acid and β-alanine according to previous reports^6,16^ with slight variations. Briefly, CDs were synthesized by mixing 1:2 molar ratio of citric acid and β–Alanine. 2.1 g of citric acid monohydrate was mixed with 1.8 g β-alanine in 10 mL water (pH3) in a conical flask. The mixture was homogenized using Ultrasonicator (Ultrasonic Cleaner FS30, Fisher Scientific, Pittsburg, PA) and heated using commercial microwave oven (1200 W#JES2251SJ02, GE Appliance, Canada) at 70% power level for 3 min to complete the carbonization and surface passivation steps. The brownish solid was then dissolved in 10 mL of distilled water.

### Purification of CDs

Purification of CDs was carried out using dialysis tubing (Spectra/Por^®^ 7 Dialysis Membrane) with 11.5 mm diameter. water was refreshed every 2 h for the first 8 h, and once a day for the next 4 days purified CDs were further sterilized by passing the solution through 0.22 µm pore-size filter^16^.

### Bacterial strains preparation

*E. coli* DH5α was cultured overnight in Luria Broth liquid medium at 37 °C and 250 rpm in shaker-incubator. The OD_600_ was adjusted by subculturing the overnight grown cells and harvested at the logarithmic growth phase. Bacterial pellet was collected from 5 ml of culture using a tabletop centrifuge and washed three times with sterile distilled water and resuspended in 1 ml of 15% glycerol. 50 µl of cells was transferred into each 0.65 ml tubes and stored at −80 °C freezer for long-term use.

### Characterization of CDs

Characterization was done according to Pandey et al., 2021^16^**.** Briefly, confirmation of functional groups of CDs was done using Fourier-transform infrared (FT-IR) spectroscopy Perkin Elmer Frontier Infrared spectrometer. Photoluminescence properties of the CDs was recorded using Synergy H1 Hybrid Multi-Mode Microplate Reader (BioTek, Winooski, VT). Zetasizer nano ZS (Malvern Panalytical Inc., Westborough MA) was used to measure the electrostatic charges of CDs.

### Binding of DNA and CDs and delivery into the cells

Two µL (3000 ng) of plasmid DNA was added into 50 µL of *E. coli* cells that were prepared earlier and 5 µL (19 mg/ml) of freshly prepared CDs was added at a later step. Samples were mixed by gentle flicking on the side of the tube. The mixture was then incubated for 15 min at room temperature. After incubation, 50 µL of water (pH 3) was added to each tube and mixture was plated (20–50 µL) on LB plates with selective antibiotic and incubated overnight at 37 °C.

### Plasmids used for study

**Figure 8:**
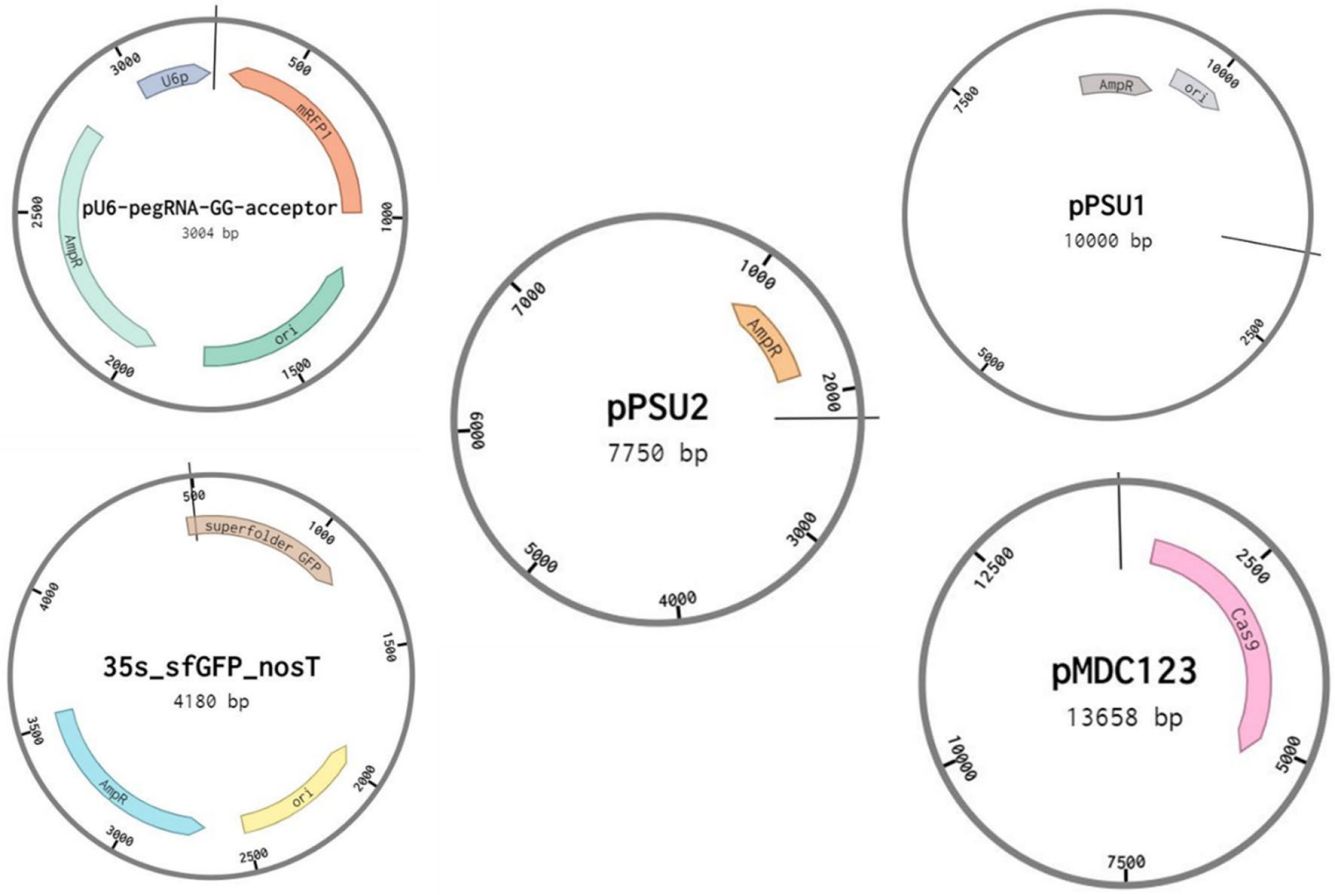
Plasmids used to evaluate the loading capacity of CDs in delivery of foreign genetic materials into the E. coli cells.

To evaluate the loading capacity of synthesized CDs, plasmids with different sizes were used for this experiment including, pU6-pegRNA-GG_acceptor (Addgene plasmid# 132777, size = 3004 bp) with RFP as reporter gene; 35S_sfGFP_nosT (Addgene plasmids# 80129 size = 4180 bp); pPSU2 (Addgene plasmid # 89566, size = 7750 bp); pPSU1 plasmids (Addgene # 89439, size = 10,000 bp) all carrying ampicillin resistance gene; and pMDC123 (Addgene # 59184, size = 13,658 bp) with kanamycin resistance gene (Fig. 8).

### Confirmation of transformation

Plasmids carrying ampicillin resistance gene, kanamycin resistance gene and RFP were used as selectable markers in bacterial transformation. Further confirmation was carried out using M13Forward and M13Reverse primers and PCR amplification of a plasmid fragment for 35S_sfGFP_nosT, pPSU1, pPSU2 and pMDC123 plasmid. Successful delivery of RFP plasmid results in red colonies that are visible without a need of fluorescence microscope.

### Effect of temperature and light on gene delivery

To evaluate the effect of incubation temperature and light on gene delivery, the mixture of cells (DH5α), plasmid (pU6-pegRNA-GG_acceptor) and fresh CDs (Citric acid + β-alanine) were incubated in 3 replicates for 15 min at different temperature including 4, 25 and 37 °C under dark or light condition.

### Statistical analysis

The number of colonies were counted using imageJ software from three replicates and analyzed using R software (V3.6.3). The mean of the treatments was calculated, and post hoc test was conducted by the least significant difference (LSD) t-test.

## Data Availability

All the data and figures are provided in the manuscript and supplementary files. Further details can be provided by reaching out to the corresponding author.
